# Abrasion of the Teeth

**Published:** 1880-02

**Authors:** 

**Affiliations:** Halle, A. S.


### ARTICLE VI.

### *Abrasion of the Teeth.*

BY DR. HERMANN, OF HALLE, A. S.

Notwithstanding all that has been written and said concerning this subject, it still remains one worthy of mention, the more so as we are yet somewhat in the dark concerning different appearances in abrasion. As for instance: defects in teeth, which cannot be struck by their antagonists, etc. The case which I will here describe has reference merely to a natural and peculiar wearing off of the teeth, with, however, singular phenomena.

A gentleman about fifty years of age, who had occasionally suffered the loss of teeth, but who bestowed much care to preserve the remaining ones, presented himself to me with two left lower teeth standing together and well worn off, both of which caused him considerable pain. They were the second bicuspid and the first molar, into the surface of which a single upper bicuspid had in the course of time, gnawed itself, and in such a manner that the depression extended equally deep in both. The teeth had long been worn off. The defect existed for many years, until finally pain in the teeth suddenly began. The pain was very intense and purely local, as the patient stated, in the lower worn-off bicuspid. By the way, let it be said that the crown of the upper bicuspid was deeply and sharply marked in the two opposite under teeth, whereas the upper one scarcely appeared to have worn off at all. The affected tooth showed no sensitiveness either at a sudden change of temperature or by scratching with the excavator on the abraided surface, nor by percussion. The pain was uniform and but seldom intermittent. I carefully examined the two adjacent teeth for caries, or if any symptoms of disease were to be discovered, in vain. The cause could only be the wearing off. I then attempted to treat with muriate of zinc, but as this would not act I repeatedly tried nitrate of silver on both teeth. It was all in vain; the patient was suffering intense agony; extraction seemed to me to be the only alternative, at least of the bicuspid, which was supposed to be the sole cause of the pain; and yet I could not resort to this action, as these teeth were the only ones with which mastication could be done, the remaining teeth not striking each other. I commenced to excavate the bicuspid below the flesh, and met, after a short time, with some success, but with so little, however, that on the following day I resolved to kill the nerve through the cavity with the usual nerve paste, which fully succeeded. On the following day the nerve was completely destroyed, and the pain temporarily ceased. By the use of a thin probe strong dentine formation was discovered established within the enclosure of the pulp cavity, toward the abraided surface. Scarcely two days elapsed when the patient again presented himself and complained of having the same pain in the adjacent molar tooth, which a few days before had its rise in the bicuspid. I made the same manipulations and met with the same success as before. An investigation again discovered new dentine formation. The patient now chews with the saved teeth as formally, and it is to be hoped will do so for a number of years yet.

The most interesting feature in the observation is probably this, that both teeth almost simultaneously showed the same phenomena. The diseased condition is most probably to be abscribed to the excessive stratification of dentine sediment and the resulting pressure on the pulpa.—*Dental Office and Laboratory.*

